# From sequence to information

**DOI:** 10.1098/rstb.2019.0448

**Published:** 2020-11-02

**Authors:** Ovidiu Popa, Ellen Oldenburg, Oliver Ebenhöh

**Affiliations:** 1 Institute of Quantitative and Theoretical Biology, CEPLAS, Heinrich-Heine University Düsseldorf, Germany; 2 Cluster of Excellence on Plant Sciences, CEPLAS, Heinrich-Heine University Düsseldorf, Germany

**Keywords:** data, sequence, information, entropy, genome, time-series, modelling

## Abstract

Today massive amounts of sequenced metagenomic and metatranscriptomic data from different ecological niches and environmental locations are available. Scientific progress depends critically on methods that allow extracting useful information from the various types of sequence data. Here, we will first discuss types of information contained in the various flavours of biological sequence data, and how this information can be interpreted to increase our scientific knowledge and understanding. We argue that a mechanistic understanding of biological systems analysed from different perspectives is required to consistently interpret experimental observations, and that this understanding is greatly facilitated by the generation and analysis of dynamic mathematical models. We conclude that, in order to construct mathematical models and to test mechanistic hypotheses, time-series data are of critical importance. We review diverse techniques to analyse time-series data and discuss various approaches by which time-series of biological sequence data have been successfully used to derive and test mechanistic hypotheses. Analysing the bottlenecks of current strategies in the extraction of knowledge and understanding from data, we conclude that combined experimental and theoretical efforts should be implemented as early as possible during the planning phase of individual experiments and scientific research projects.

This article is part of the theme issue ‘Integrative research perspectives on marine conservation’.

## 1. Introduction

When discussing the process of generating useful information from sequences, it is helpful to agree on some basic definitions. First, we need to clarify what exactly we consider a sequence and what we understand as information. When speaking about sequences, most biologists understand a sequence found in biological macromolecules, such as the sequence of nucleotides within a DNA or RNA molecule or the sequence of amino acids within a protein. Strictly speaking, sequences are far more general and describe any set of objects (real: such as chemical compounds, or abstract: such as numbers) arranged in some sequential order. In this work, we will mostly refer to biological sequences given by the order of chemicals arranged in a sequential order within a macromolecule, but would like to stress that measurements obtained at various time points also represent a sequence, from which plenty of useful information can be extracted. Such sequences were in particular important before the advent of high-throughput technologies that allow macromolecular sequences to be read efficiently. As we will discuss, sequences of sequences, i.e. time-series of biological sequence data, are a valuable method to infer information from sequences.

While sequences are rather straightforward to define in a very general sense, it is far more challenging to capture the notion of information in a simple definition. In information theory, information—or rather the generation of information—is quantified by the information entropy (or Shannon entropy, named after Claude Shannon who introduced the concept in 1948 [1]). The concept of information entropy is highly useful to determine, for example, bounds for lossless compression, and helps in quantifying the capacity of transmission systems to transmit data.

The difficulty is that in this theory data are inherently considered to be identical with information, and the encoding and decoding processes during communication are concerned primarily with the problem of encoding, transmitting and decoding a sequence of bits—the fundamental unit of information. The important question whether the receiver actually understands the transmitted information is not considered in this theory at all.

It is very simple to calculate the Shannon entropy of an arbitrary text, and the resulting number will tell us how randomly (or non-randomly, and thus *surprisingly*) the letters are arranged into a sequence. However, the same information (for example as contained in a user manual of a microwave or any other technical device) can be written in many languages. The Shannon entropies of all these texts may be the same, or at least very similar. But for me as a receiver it makes a great deal of difference whether the text is written in English (which I understand) or in Finnish (which I don’t). This example illustrates that the information content of data, as quantified by the Shannon entropy, does not help us to predict how much useful information we can extract. It further illustrates that, in addition to the data themselves, knowledge about the decoding system (here, knowledge of a language) is required to actually make use of the information. In the following, information is interpreted as ‘knowledge obtained from investigation, study, or instruction’,^1^ which entails that besides the pure information content also the associated decoding mechanisms are considered.

**Figure 1.**
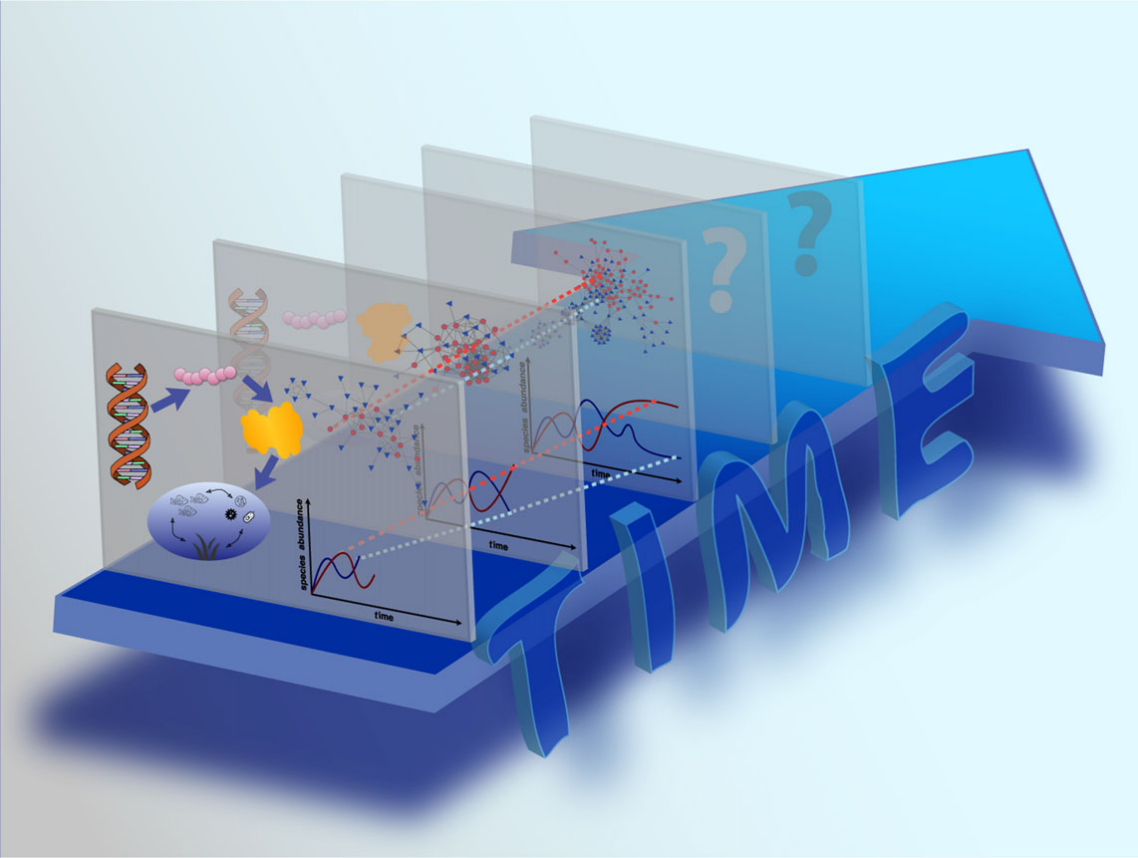
From sequence to information. This figure shows the different levels of information, from DNA to environment. Each layer depicts a different level of information that can be obtained from sequences. The DNA sequence encodes the genetic information that is decoded by the translational machinery into amino acid sequences. These in turn fold into functional proteins. The protein functions provide information about the capabilities of an organism such as its metabolism. Combined information of many organisms and environmental parameters characterize ecosystem dynamics. All these information layers can be used to infer different relationships, for example, in the form of networks or models. Including the temporal aspect (big blue 3D arrow), another dimension of information is gained, from which temporal correlations and interactions can be determined. A major task of time-series analysis and mechanistic modelling is to predict the future from information collected from the past. The more distant the future is that we try to predict, the more the uncertainty (question marks) increases.

Our text is structured as follows. First, we survey which information is contained in various biological sequences, and illustrate how the information content changes when considering different levels of biological organization. We would like to note here that there exist a much higher number of biological organization levels than we address in this article, and we have selected the most fundamental ones to briefly exemplify this process. Further, we outline how information is transmitted and decoded, and discuss what kind of useful information, or *knowledge*, can be obtained from the data. We then proceed towards time-series data, which, as mentioned above, also represent a sequence containing useful information, and illustrate how new knowledge and insight are produced by different types of analysis. The multiple layers encompassing different information content are illustrated in figure 1. We conclude by suggesting that experiment and theory need to collaborate more intensely, and that this collaboration, and in particular interdisciplinary communication, need to be implemented as early as possible, during the planning and experimental design phase.

## 2. Information in biological sequences

All life on Earth is based on genetic sequences stored in DNA. These sequences contain key information on how to manufacture and assemble the building blocks composing an organism, how to regulate the activity of various components in response to the environment, and, most importantly, how to copy this information and transmit it to future generations. Copying information is never perfect, so information can be changed and reassembled in different combinations.

Passing the information from ancestor to descendant, or laterally between organisms, while at the same time modifying it through random mutations, inevitably led to speciation [2–4], which resulted in the enormous biodiversity on this planet. Analysing the information stored in the genetic material is a first step of a comprehensive investigation of the processes required to extract and decode biological information.

### (a) DNA

Understanding information as a signal that becomes valuable after decoding by a receiver, a DNA sequence contains more informative content than the sequence of the four different nucleotides that a DNA molecule is composed of.

The order of the nucleotides within the DNA sequence reduces the information entropy. In eukaryotes for example, the genome sequence contains several types of repeated nucleotide sequences (repeats). This phenomenon results in a reduction of the DNA information entropy, as was shown in an earlier study [5]. As a beneficial result, repetitive nucleotide sequences provide genetic redundancy and gene regulation by DNA folding specificity, and are important for the synthesis of proteins with similar functions [6, p. 556]. However, at the same time the order of nucleotides increases the complexity of information storage [7]: it is responsible for the helix structure, which itself affects the robustness [8] of the double helix or the accessibility [9,10] of the DNA sequence for the interaction of organic compounds or inorganic nanomaterials [11]. For example, measuring the periodicities of 10–11 bp allows the super-coiled state of genomic DNA to be determined [12–14]. Supercoiling illustrates how sequential information stored in DNA base pairs can be translated into structural information about the DNA molecule. DNA supercoiling strongly affects DNA metabolism, has influence on the molecular evolution of the DNA [15] and is one of the most fundamental regulators of global gene expression in bacteria [16,17]. The next level of coiled DNA ordering is the specific chromosome structure, which defines almost the whole library of inherited genetic information of an organism. A disorder of this information level can cause damage to a biological system, for example the duplication of one chromosome in humans (e.g. trisomy 21) results in several health problems [18]. Information stored in the non-randomly ordered nucleotide triplets (codons) [19–21] forms the basis for the genetic code. Only this code allows DNA sequences to be scanned, decoded and interpreted by the translational machinery, to be converted into amino acids in a process that enables relocating inherited information into proteins, another set of elementary biological buildings blocks. The genetic code is perhaps the most illustrative example for the fact that yielding useful information from data always requires a functioning data decoding system. Interestingly, this information transfer from DNA to protein is highly dynamic. For example, identical proteins can be synthesized with different molecular energies if the same amino acid sequence is encoded by different codons [22].

### (b) Genes

Proteins, defined by the information encoded in the DNA sequence (the gene), fulfil certain functions within a living organism. Information gathered from specific marker genes allows conclusions about evolutionary forces that are responsible for adaptation and speciation processes. For example, the most commonly used marker gene in prokaryotes is 16S ribosomal RNA (rRNA) [23]. Because this gene is considered to have an essential function, it is ubiquitous, and it exhibits a low mutation rate, comparative analyses of the DNA sequences allow reconstruction of the evolutionary history of species. Such phylogenetic reconstructions can identify clades specific to certain ecosystems, such as the SAR11 clade [24], or some archaeal species that have been identified in the euphotic zones of marine ecosystems [25]. However, interpreting results based on marker genes like 16S rRNA and thus extracting accurate information are complicated by various factors [26,27], including the experimental amplification bias, as shown by Hong *et al.* [28], or its presence in multiple copies [26]. Alternative single-copy markers like chaperonin-60 [29] or the *rpoB* gene provide more phylogenetic resolution than the 16S rRNA gene and are often used in gathering evolutionary information [23].

Proteins resulting from the translation of the DNA sequence may, in the simplest case, perform exactly one function. However, there are multiple known examples where this simple one-to-one relation is not accurate. Multifunctional proteins, the so-called ‘moonlighting proteins’, perform more than one biochemical or biophysical function [30,31]. Protein moonlighting means that a gene may acquire and maintain a second function without gene duplication and without loss of the primary function. As a result, such a gene is under two or more entirely different selective constraints [32]. In a nutshell, we observe that the information stored in a gene sequence is much larger than is recognized by standard comparative methods. Therefore, the optimal yield on the information stored in sequences is best obtained by the agglomeration of different research methods. Wrapping particular experimental studies in the laboratory with theoretical predictions obtained from mathematical and statistical analyses is one promising path forward to maximize the information extraction process.

### (c) Genome

Zooming out from the level of single genes to the whole library of genes stored in an organism’s genome allows extraction of information from the sequence in a different context. Considering the whole genome as information source, several sequence characteristics can be scanned to coax out functionality encoded in the genome structure. Focusing on the GC content variation between organisms, for example, points to genomic adaptations that might have played a significant role in the evolution of the Earth’s contemporary biota [33]. In addition, genomic GC comparison allows identification of recombination events that are responsible for shaping the information flow along the genomes in an evolutionary context [34–36]. Besides the specific distribution of the nucleotides within a genome sequence, the order of genetic blocks itself entails information that is decodable and allows conclusions about mechanisms that are responsible to populate the genome with new information. Genome synteny analysis (the relative gene-order conservation between species) can provide key insights into evolutionary chromosomal dynamics and the rearrangement rates between species [37–40]. Today, information based on complete genome sequences is mainly obtained by comparative genomic approaches. Investigation methods focusing on the pan-genome (genes present in all strains) [41] of a species elevates information mining to a new perspective. Pan-genome studies allow investigation of the plasticity of a genome at species level. For example, insertions, deletions, and recombination events, as well as single-nucleotide polymorphisms (SNPs), are only visible at pan-genome level, highlighting the consequences of evolutionary forces [41–45].

### (d) Gene expression

Whereas the genomic content stored in the DNA remains rather constant throughout the lifespan of an organism, the rates with which individual genes are transcribed vary strongly over time. Transcription is regulated by multiple factors, including environmental stimuli. The result of this regulation can be observed by measuring the quantity of the messenger RNA transcripts (mRNA) under different conditions or over time. These data provide additional information that cannot be obtained from the DNA sequence alone. Transcriptomics techniques allow analysis of the entirety of all transcripts available from one organism in different tissues, under different conditions or at different time points. Information obtained by transcriptomics allows conclusions about the regulation of gene expression. There are two key contemporary techniques in the field: use of microarrays, which quantify a set of predetermined sequences, and RNA sequencing (RNA-Seq), involving high-throughput sequencing to capture all sequences [46]. For medical applications, expression data have been successful in providing a molecular basis for the diagnosis of otherwise difficult to distinguish pathologies [47–49]. In addition, co-expression profiles analysed using network and machine learning approaches [47,49,50] helped to discover functionally linked genes that are associated with specific diseases [51]. In microbiological research, co-evolutionary aspects of bacteria and their viruses (phages) are an impressive example where gene expression analysis helped in understanding the mechanistic interactions in greater detail [52,53]. Co-expression analysis is not limited to a specific genome, but can be also observed among different species, where it displays remarkably similar synchronous patterns of gene expression over time [54]. Nevertheless, the expression profile extracted and evaluated by common methods will not necessarily provide information about interactions between genes or proteins. For example, the two-component signal transduction system in bacteria is able to recognize and respond to a variety of environmental stimuli. This basic system is composed of a sensor histidine kinase that catalyses its autophosphorylation and subsequently transfers the phosphate group to a response regulator, which can then trigger different physiological changes [55–57]. This regulatory mechanism cannot be understood just by scanning the information written in the genetic sequence nor by studying its expression profile. This complex mechanism is best explored by experimental work that can be integrated in the framework of mathematical models [58–60]. Such combined interdisciplinary approaches have been successfully applied to the analysis of the MAP kinase pathway, which was performed by a combination of mathematical modelling, integrated phosphoproteomic technology and Western blotting [61]. In summary, gene expression analysis is a major contributor for our understanding of gene regulation.

### (e) Functional profiling

One of the main goals of sequence analysis is the determination of functional properties. The corresponding methods are often referred to as ‘functional profiling’. This process usually begins by comparative analyses of sequences of interest with annotated databases. For instance, after sequencing the protein coding gene of interest, the obtained reads are mapped on a reference database like the Kyoto Encyclopedia of Genes and Genomes (KEGG) orthology [62–64], Clusters of Orthologous Groups (COGs) [65,66], Non-supervised Orthologous Groups (NOGs) [67], Pfam [68] and UniProt Reference (UniRef) clusters [69]. These databases are used in order to classify only protein coding sequences into a putative functional category. Efficient search methods like BLAST [70] provide a putative classification into a functional category through sequence similarity. For the assumption that similar sequences perform similar functions, this approach is very successful if a reference protein or gene/genome exists.

If the same function is encoded in highly identical protein sequences, then we would consider the information entropy of such sequences in general as very low. Sometimes sequences may perform the same function but are different in their content, e.g. in amino acid compositions. An example is the LSR2 protein, which is a transcriptional silencer found in Actinobacteria, where it binds AT-rich DNA and silences its transcription [71–73]. This example illustrates how information stored in a sequence can drastically differ depending on the level of organization that is considered: the information entropy based on the arrangement of the amino acids in the sequences is extremely high, which results from the diversity between the sequences. On the other hand, the same sequences exhibit a low information entropy when their functional properties are considered. This can be best observed when the secondary structure of the sequences is considered [71].

Functional profiling of genes and proteins is an important step in understanding the role of a sequence in the context of the whole genetic repertoire of an organism. How genes interact on the functional level is yet a higher level of information, from which new knowledge can be extracted.

### (f) Pathway reconstruction

Understanding biological systems presupposes investigating how matter and energy are converted in order to maintain their functions. How exactly these processes work is very likely written in the genetic sequence. To decode it, we need more understanding than the information from sequence content alone, or how strong a gene is expressed. Rather, the interplay between various gene functions is essential. Metabolic pathway reconstruction, molecular interaction and reaction network analysis, followed by mapping processes to reference pathways, increase our understanding about a higher-level function of an organism [74–76]. Once a reaction network has been reconstructed, it can be analysed using various structural analysis techniques, such as the method of network expansion [77], or dynamic approaches based on ordinary differential equations (ODEs) [78,79]. Such approaches allow us to systematically investigate the effect of changes in parameters that are not easily accessible experimentally, and thus to draw general conclusions about regulatory principles [80–82]. In addition, two more promising concepts for pathway analysis that assesses inherent properties in biochemical reaction networks [83,84] rely on the related concepts of elementary flux modes [85,86] and extreme pathways [87–90]. Pathway analysis undoubtedly has great potential to gain a better understanding of cellular metabolism. For example, the potential of micro-algae to uptake large quantities of phosphorus (P) and to use it as biofertilizer has been regarded as a promising way to redirect P from waste water to fields. This also makes the study of molecular mechanisms underlying P uptake and storage in micro-algae of great interest [91]. Pathway reconstruction efforts in general uncover dynamic processes that take place at cellular level and are written down in the genetic code by an evolutionary process subject to environmental adaptation pressures. Considering additional information by including environmental parameters is a necessary step towards a comprehensive understanding of the ecological processes including niche adaptation.

### (g) Meta-omics: what information is there?

Fundamental research in biology heavily relies on model organisms. They have been used to uncover mechanisms that synthesize, modify, repair and degrade the genetic sequence and its encoded product, the signalling pathways that allow cells to communicate, the mechanisms that regulate gene expression and the pathways underlying diverse metabolic functions [92–96]. In order to describe many aspects of information retrieved from sequences obtained at specific time points and/or conditions, or to understand the evolutionary history of non-cultivable organisms, ‘omics’ data-integration techniques are essential [97,98]. Meta-omics pools the knowledge of how to read and decode the information from a sequence, as described in the previous paragraphs, together with environmental parameters that are collected with the sequences. High-throughput ‘omics’ techniques allow observation of metagenomes, metatranscriptomes and proteomes and thus are important to describe the behaviour of populations of uncultured microorganisms and give hints on their population genetics and biogeochemical as well as ecological interactions, which cannot easily be studied or modelled in laboratory systems [99]. High-throughput DNA sequencing enabled investigation of diverse environmental and host-associated microbial communities, thus identifying for example several new virophages [100,101] or even discovering completely new prokaryotic phyla [102]. The discovery of the Asgard superphylum, a group of uncultivated archaea including the Loki-, Thor-, Odin- and Heimdallarchaeota, and the proteins with similar features to eukaryotic coat proteins involved in vesicle biogenesis, which are present in this phylum, altered significantly our understanding of the origin of life [102,103]. These organisms were isolated from marine sediments that were sampled near Loki’s Castle (a field of five hydrothermal vents that are located in the middle of the Atlantic Ocean between Greenland and Norway) [104].

Metatranscriptomics allows researchers to quantify community gene expression in an environmental sample using high-throughput sequencing technology. Today we have several pipelines (e.g. SAMSA2) to analyse the huge amount of data efficiently using high-performance computational utilities [105]. Such analyses enable quantification of gene expression and its regulation within multiple organisms in order to derive conclusions about specific molecular interactions [106]. Combining metatranscriptome and gene sequencing with time-series design allows us to collect information about the dynamics of different organisms in an environmental context [107].

Metaproteomics (community proteomics) characterizes all the proteins expressed at a given time within an ecosystem. This allows us to create hypotheses and draw conclusions about microbial functionality. Further it makes it possible to study the adaptive responses of microbes to environmental stimuli or their interactions with other organisms or host cells [108–110]. Analysis of communities in natural environments has contributed immensely to our knowledge of microbial functions, such as nutrient cycling, mutualistic endosymbiosis, organic matter degradation, metal utilization and eutrophication response [108,111–113]. Despite the additional information that is gathered through ‘omics’ analysis, understanding biological processes as a whole is incomplete without considering their dynamic aspects. Therefore, only by including a temporal dimension will we be able to understand and model bio-ecological processes in detail [114].

## 3. Ecosystem dynamics: time-series analysis

Most methods reviewed above extract and study information from genomic sequences, either alone or in a comparative context, but mostly as static structures without considering any temporal dynamics. Gene expression information describing the quantity of reads obtained either in different conditions or from different time points does contain time as a factor. Whereas comparative genomics can generate hypotheses regarding the evolutionary dynamics of genes and genomes, dynamics on shorter time-scales have not yet been discussed. It is apparent that even the best meta-omics dataset obtained for a single time point cannot yield any information regarding, for example, the mechanisms underlying the population dynamics observed in an ecosystem. Before we discuss recent and ongoing approaches to analyse time-series of sequence data and extract mechanistic information, and thus understanding, we briefly summarize essential concepts of time-series analysis in general.

The main objective of time-series modelling is to carefully collect and examine observations from the past in order to develop a suitable model that describes the inherent structure of the series. This model is then used to generate future values for the series, i.e. to make predictions [115]. The prediction of time-series can therefore be described as the process of predicting the future by understanding the past.

There are many ways to analyse time-series data, depending on how much prior knowledge is available about the underlying mechanisms. Often we first distinguish between seasonal, cyclic and irregular components [116]. Analysing seasonal changes in the diversity of bacterial communities [117] has, for example, suggested that seasonal changes in environmental variables are more important than trophic interactions. Cyclic fluctuations describe recurrent medium-term changes. The metagenome data of Biller *et al.* [118] contain, for example, genomic information for a large number of bacteria, archaea, eukaryotes and viruses. The usefulness of the data is enhanced by the availability of extensive physical, chemical and biological measurements associated with each sample. In this way, the different cyclic changes within the habitats could be investigated and possible causes identified.

When adapting a model to a dataset, particular attention should be paid to selecting the most economical model. Here, ‘most economical’ refers to the simplest possible model that can explain the data without overfitting [116]. One of the most popular and commonly used stochastic time-series models is the Autoregressive Integrated Moving Average (ARIMA) [119]. This model considers each time-series as a collection of linear approximation and the deviations from these fits needs to follow a statistical distribution, representing the noise. ARIMA's popularity is mainly due to the flexibility to represent several types of time-series in a simple way. There are many examples of how to use the ARIMA model [54,120–123]. An example are the effects of starfish wasting diseases in the Salian Sea, a Canadian–American border area, a marine ecosystem and global hotspot for the biodiversity of temperate asteroids with a high degree of endemism [120]. Species- and area-specific ARIMA models and their estimated parameter values showed that after the outbreak of the starfish wasting disease epidemic in 2013 the incidence of the starfish *Dermasterias imbricata* increased in three areas. The observed frequency of *D. imbricata* until 2015 exceeded the model prediction for population development. The serious limitation of the model, however, is the assumed linear form of the associated time-series, making it insufficient in many practical situations.

A commonly applied methodology for the investigation of nonlinear stochastic models is the use of artificial neural networks (ANNs). Their characteristic is the application to time-series prediction problems by their inherent ability to nonlinearly model without having to adopt the statistical distribution. The corresponding model is formed adaptively on the basis of the specified data. For this reason, ANNs are inherently data-driven and self-adaptive. The most common and popular are multi-layered perceptrons (MLPs) characterized by a single feed-forward network (FNN) with a hidden layer. This method has a wide range of applicability. For example, phage protein structures could be predicted based on the genetic sequence [124]. In a different context, the functional roles of interacting microbes could successfully be predicted from environmental parameters and intramicrobial interactions [125].

## 4. Mechanistic models

The strategies to analyse time-series data discussed above are essentially statistical methods that aim at extracting patterns from time-series without using prior knowledge in order to make predictions about underlying mechanisms. Mechanistic models pursue a complementary approach. Based on experimental observation and often a great deal of intuition, a researcher formulates hypotheses on certain underlying interactions that give rise to an observable macroscopic behaviour. These hypotheses are then translated into equations capturing the interactions in a quantitative way. Solving these equations generates simulation results that can be compared with experimental observations, thus verifying or falsifying the initial hypotheses. This approach has been extremely successful for relatively small systems and for very fundamental questions. Almost a century ago, Lotka [126] and Volterra [127] independently developed a simple mechanistic model of two interacting species that demonstrated how oscillations in populations of a predator and a prey species can be explained as an emergent property from simple underlying mechanistic assumptions. Not surprisingly, the Lotka–Volterra model forms the basis for a multitude of more complex models and serves as a foundation to study fundamental questions, such as the conditions for co-existence of species [128]. Generalizing the ideas and equations of Lotka and Volterra leads to the class of generalized Lotka–Volterra (gLV) models, which are commonly used to study the dynamics of ecosystems [81], including the dynamics of bacterial communities [129,130]. Whereas gLV models only contain the interacting species as variables and thus define direct interactions between species, consumer resource models developed by MacArthur [131] also consider the resources as variables. Most recently, these models have been employed to explain which environmental factors determine the species richness, i.e. the number of species that can co-exist in an ecosystem [132,133]. When the first dynamic ecosystem models were developed early during the twentieth century, no information on biological sequences was available. However, the data triggering the theories of Lotka and Volterra were time-series, i.e. sequences of estimated numbers of predator and prey species, such as the data on numbers of pelts collected by the Hudson’s Bay Company [134]. Now, the question arises how time-series of biological sequence data can be employed to construct mechanistic models that generate understanding about the underlying mechanisms guiding the temporal evolution of an ecosystem.

Owing to the high throughput and the resolution, time-resolved 16S barcoding data contain information on hundreds of species. Barcoding is referred to a global bioidentification system that employs DNA sequences as unique identifiers linked mostly to a specific taxonomic unit [135]. Deriving mechanistic models from barcoding time-series was illustrated for example by Stein *et al.* [136], who developed a modified gLV model that correctly predicted the community composition of the intestinal microbiome of mice under different conditions. Based on barcoding data describing the bacterial community associated with the marine diatom *Phaeodactylum tricornutum*, Moejes *et al*. [137] demonstrated that four bacterial families dominate the phycosphere, and development of a consumer resource model illustrated the high degree of uncertainty in deriving mechanistic explanations from time-series abundance data, especially if the time resolution is low.

Genomic sequence, together with functional annotation, allows the reconstruction of genome-scale metabolic network models, which encompass the complete biochemical repertoire encoded in an organism’s genome [138]. The most commonly used technique to analyse such models is flux-balance analysis (FBA) [139], which allows calculation of internal flux distributions and nutrient exchange rates for given external conditions under the assumption that the metabolism is configured in order to optimise a certain objective function, such as maximising the accumulation of biomass [140,141]. With these and related metabolic network analysis methods, such as elementary flux mode analysis [86] or the method of network expansion [77,142], it became possible for the first time to rigorously link the genotype to the phenotype, where of course the view is centred on metabolism alone [143]. Not surprisingly, the enormous power that genome-scale modelling approaches provide led to an integration of such approaches in a dynamic context. Dynamic FBA (dFBA), for example, uses the flux predictions resulting from FBA at a given time point to dynamically update nutrient and biomass concentrations [144]. This approach was successfully employed to explain and predict the dynamics of interacting organisms and their environment [145].

The current development of modelling techniques to simulate interactions of organisms on a metabolic level proceeds with enormous momentum. Controlled mesocosm experiments [146] allow for controlled environments, in which not only the community dynamics and the temporal expression patterns can be measured, but also the micro- and macronutrients as well as cofactors in the bulk solution can be determined to derive a deeper understanding of the metabolic interdependencies within microbial communities. This clearly illustrates the key role controlled environments play in rigorously testing and improving new hypotheses and theories.

## 5. Conclusion

The key question for the future is how can we ensure that ongoing data collection efforts, generating vast amounts of biological sequence data, are optimally suited for the development of mechanistic models. These cannot only describe data, but also rationalize what we observe based on underlying fundamental mechanisms. It is understandable that, when a new and rather unknown system, such as the global marine microbiome, is investigated for the first time, a rather unbiased, exploratory approach is taken, as is exemplified by the *Tara* Oceans expedition [147]. The enormous mass of sequencing data is certainly useful, because it provides us with an inventory of genes that are found in marine microbes. Moreover, by combining sequence data with physical parameters and metadata, novel hypotheses can be generated, such as a functional dependence of species richness and water temperature [148]. Despite the size of the generated data resource, it still only describes a snapshot of microbial abundance, albeit with considerable detail. Thus, the information gained from the data is mostly restricted to observing what is there. It is hard to conceive that the dataset would allow answering of fundamental scientific questions, such as those regarding the underlying mechanisms guiding microbial ecosystem dynamics. It is plausible to assume that for such an endeavour a more targeted approach is required. For example, to collect barcoding, metagenome and metatranscriptome data with a high temporal and spatial resolution may be a constructive way forward towards testing specific hypotheses regarding the mechanisms by which key microbial species interact. Such experiments can be designed following some successful research guidelines from this field [149–151].

This example demonstrates that the amount of data does not necessarily correlate with the gain of basic understanding. In other examples, such as the dynamics of the phycosphere of *P. tricornutum* [137] in controlled environments, we clearly have too little data to test the numerous existing hypotheses about the mechanistic interactions between species. By discussing the information content within biological sequences and the information flow between various levels of biological organization (e.g. amino acid sequence and corresponding secondary structure), we have shown that extracting knowledge from different information layers can be more effective and fruitful for sequence data analysis. For example, in order to analyse the abundance and diversity of silencer proteins in several environments a simple comparative sequence analysis will fail owing to the high information entropy at the amino acid level. Therefore, machine learning approaches using the information stored at the different levels need to be considered as a first step of the analysis pipeline to predict putative candidates from different metagenomic datasets. In a second step, laboratory experiments need to be performed, like DNA affinity chromatography followed by ChAP-Seq analysis. This combination of computational work and laboratory experiments should highlight how important the theoretical envelope for laboratory study becomes when information from different levels is considered.

We conclude that two main aspects will become increasingly important for biological research in the near future to close the gap that currently exists between the vast amount of high-throughput data and the actual fundamental understanding generated from it. Firstly, methods need to be developed, and already existing ones need to be implemented in the daily experimental work process and refined to integrate different types of data. Today several methods already exist for particular sequence analysis [152–154]. A minority are available for time-series implementation on biological data [155–157]. This refers primarily to the integration of time-resolved sequencing data with meta-information describing external conditions like pH, temperature, dissolved oxygen, CO_2_, phosphate, nitrate, salinity, pressure, chlorophyll density, etc. Moreover, novel approaches will be required to integrate results from different methods of data analysis to maximise the information gain. Secondly, after an era of mainly exploratory data acquisition, it is of paramount importance to strengthen hypothesis-driven experimental approaches [158,159]. Every research question requires its own special experimental treatment. The prevailing misconception that data acquisition comes before (and is separated from) model development often leads to a design of research projects in which interdisciplinary collaborations are restricted to the data analysis phase. In our opinion, these flaws in project design lead to inefficiency and a sub-optimal coordination between experiment and theory. As an example of how theoretical knowledge supports experimental biology, and moreover enables new insights into biological processes, we mention the studies by Marsland *et al.* and Goldford *et al.* [132,133,160]. Bridging theory and experiment, in their studies the authors monitored the assembly of hundreds of soil- and plant-derived microbiomes in well-controlled minimal synthetic media. The resulting communities were sequenced using 16S ribosomal RNA, and the outcomes were modelled mathematically. Their mathematical models could reproduce large-scale ecological patterns observed across multiple experimental settings [133].

In fact, we are convinced that the involvement of theory cannot begin too early. Bioinformaticians and modellers should be involved during experimental design, because these researchers are typically those that formulate clear working hypotheses and have a model structure in mind, even before a detailed mathematical model has been constructed. Only in close interdisciplinary discussion can the different goals and aims of experimentalists and theorists be harmonized, and experiments be planned so that the resulting data are optimally suited to build mechanistic models and test scientific hypotheses.

## Endnote

1 Merriam-Webster Online Dictionary, https://www.merriam-webster.com/dictionary/information, retrieved 8 November 2019.

## Data accessibility

This article has no additional data.
